# Bipolar risk and mental imagery susceptibility in a representative sample of Chinese adults residing in the community

**DOI:** 10.1177/0020764015597951

**Published:** 2016-02

**Authors:** Roger Man-kin Ng, Stephanie Burnett Heyes, Freda McManus, Helen Kennerley, Emily A Holmes

**Affiliations:** 1 Department of Psychiatry, Kowloon Hospital, Kowloon, Hong Kong; 2 School of Psychology, University of Birmingham, Birmingham, UK; 3 Department of Experimental Psychology and Department of Psychiatry, University of Oxford, Oxford, UK; 4 Oxford Cognitive Therapy Centre, Warneford Hospital, Oxford, UK; 5 MRC Cognition and Brain Sciences Unit, Cambridge, UK; 6 Department for Clinical Neuroscience, Karolinska Institutet, Stockholm, Sweden

**Keywords:** Mental imagery, Chinese adults, bipolar disorders, Hong Kong, hypomania

## Abstract

**Background:**

We need to better understand the cognitive factors associated with risk for bipolar disorders. Recent research suggests that increased susceptibility to mental imagery may be one such factor. However, since this research was primarily conducted with Western students and at a single time-point, it is not known whether the relationship between imagery susceptibility and bipolar symptoms exists across cultures or within the general community, or whether this relationship remains stable over time.

**Aim:**

This study evaluated whether Chinese adults identified as being at high (HR) versus low (LR) risk of developing bipolar disorders showed greater mental imagery susceptibility. We aimed to test whether such a relationship was stable over time by measuring imagery characteristics at baseline and at the 7-week follow-up.

**Method:**

This prospective study recruited a community sample of *N *= 80 Chinese adults screened for the absence of neurotic and psychotic disorders. The sample was split into HR (*n *= 18) and LR (*n *= 62) groups at baseline based on a criterion cut-off score on a measure of hypomania, the Mood Disorder Questionnaire (MDQ). Participants completed measures of imagery susceptibility and its impact: the Spontaneous Use of Imagery Scale (SUIS) and the Impact of Future Events Scale (IFES), at baseline and 7 weeks later.

**Results:**

HR group reported greater tendency to use imagery in daily life (SUIS) and greater emotional impact of prospective imagery (IFES) than LR group at baseline. These results remained stable at follow-up.

**Conclusion:**

This study provides preliminary evidence for increased susceptibility to mental imagery in individuals at high risk of bipolar disorders recruited from a community sample of Chinese adults. This extends previous research in Western student samples suggesting that imagery (both levels of use and its emotional impact) may be a cognitive factor with cross-cultural relevance that is stable over time.

## Introduction

Bipolar disorder is a serious and recurrent disease with substantial disability (Murray & Lopez, 1997). Lifetime prevalence of bipolar disorders is 4.4% if hypomania and other minor elated states are included in the definition (Merikangas et al., 2011). Minor elated states could be conceptualised as the severe end of the risk continuum (Beesdo et al., 2009) and are reliably detected among the general population using questionnaire measures of hypomania such as the Mood Disorder Questionnaire (MDQ; Deeprose, Malik, & Holmes, 2011; Hirschfeld et al., 2003; Malik, Goodwin, Hoppitt, & Holmes, 2014; Rock, Chandler, Harmer, Rogers, & Goodwin, 2013). Previous studies have suggested that minor elated states confer an enhanced risk of full-blown bipolar disorders (Tijssen et al., 2010) and are associated with increased comorbidity for depression and impulse control problems (Rock et al., 2013). Understanding the factors associated with bipolar risk states might therefore be of public health significance. There has been a recent call for research investigating the contribution of cognitive and emotional factors associated with risk for bipolar disorders (Johnson, 2005). One such potential cognitive factor is mental imagery susceptibility (Holmes, Geddes, Colom, & Goodwin, 2008; Ng, Krans, & Holmes, 2013).

Mental imagery is characterised by a subjective resemblance to sensory impressions, as if ‘seeing with the mind’s eye’ or ‘hearing with the mind’s ear’ (Kosslyn, Ganis, & Thompson, 2001). Due to its powerful impact on emotion, mental imagery has been postulated to play a key role in maintaining emotional psychopathology (Holmes & Mathews, 2010). In Holmes et al. (2011), patients with bipolar disorder experienced greater use of imagery in their daily life, compared to non-clinical controls, and were more emotionally affected by future-oriented imagery (i.e. prospective imagery). In another study, patients with bipolar disorder reported greater use of imagery in daily life than did patients with unipolar depression (Hales, Deeprose, Goodwin, & Holmes, 2011). In patients with bipolar disorder, the emotional impact of prospective imagery predicted self-reported levels of drive within the behavioural approach system (BAS) postulated to be closely associated with the risk of mania (Ivins, Di Simplicio, Close, Goodwin, & Holmes, 2014).

It has been suggested that mental imagery contributes to emotional amplification of both manic and depressive symptoms among euthymic patients with bipolar disorder (Hales et al., 2011; Holmes et al., 2011; Holmes et al., 2008). However, in order to evaluate whether mental imagery is associated with vulnerability to bipolar disorder, behavioural risk studies are needed (Riskind & Alloy, 2006). In such studies, participants are recruited who exhibit certain cognitive and behavioural vulnerabilities thought to create liabilities for bipolar disorder, without meeting the full criteria for the disorder (Just, Abramson, & Alloy, 2001). In one recent study, a non-clinical student sample divided into high (HR) and low (LR) bipolar risk groups based on a criterion cut-off score on the MDQ completed the Impact of Future Events Scale (IFES; Deeprose & Holmes, 2010). Results showed that the HR group experienced greater emotional impact of prospective imagery, and a greater number of negative prospective images, than the LR group (Deeprose et al., 2011). In Malik et al. (2014), participants at greater risk of developing bipolar disorders (according to the same MDQ criterion) showed greater tendency to use imagery in daily life, as measured by the Spontaneous Use of Imagery Scale (SUIS; Reisberg, Pearson, & Kosslyn, 2003).

While these findings are potentially valuable, the studies suffer from a number of limitations. First, Deeprose et al.’s (2011) sample did not undergo any formal screening for the presence of psychiatric disorders. Second, in previous studies, the HR group showed higher levels of current depressive and/or anxiety symptoms than the LR group. Since distressing imagery has also been reported in patients with anxiety and depressive disorders (Morina, Deeprose, Pusowski, Schmid, & Holmes, 2011; Patel et al., 2007), the presence of such symptoms might confound putative relationships between mental imagery susceptibility and bipolar risk. Third, previous studies did not rule out psychosis proneness, which has been associated with greater pre-living of imagined events (Winfield & Kamboj, 2010). Fourth, the samples comprised mostly students from the United Kingdom, and the extent to which findings will generalise across age groups and cultures is unknown (Perlis et al., 2004; Poon, Chung, Tso, Chang, & Tang, 2012; Schurhoff et al., 2000). Fifth, previous studies administered imagery measures at a single time-point. Whereas mental imagery susceptibility has been assumed to be stable in the absence of changes in mood or neurotic symptoms, this assumption has not been directly examined in a prospective study. This study was conducted to address these limitations.

This study aimed to replicate and extend the findings of Deeprose et al. (2011) and Malik et al. (2014) by recruiting a representative community sample of ethnic Chinese adults assessed to be free from psychotic or neurotic disorders. Participants categorised as being HR versus LR according to the MDQ completed imagery measures twice, 7 weeks apart. This 7-week follow-up interval was selected as initial pilot data in *n *= 20 health care professionals indicated 4-week test–retest stability of the IFES, suggesting the IFES might measure a trait-like imagery characteristic. We extended this period to examine stability of the IFES over a period of 7 weeks, while minimising the risk of dropout with longer follow-up periods. Mood symptoms in bipolar disorders can fluctuate severely over this time period (Bonsall, Wallace-Hadrill, Geddes, Goodwin, & Holmes, 2012). Therefore, if imagery characteristics do relate to bipolar risks but do not fluctuate with time, this would be of interest.

Hypotheses were as follows:

1. Compared with the LR group, the HR group would show greater imagery susceptibility as assessed using the IFES and the SUIS.
2. This pattern of characteristics would remain stable over time – that is, higher in the HR than LR group at the 7-week follow-up.

## Methods

### Study design and participants

Participants (*N *= 80) were recruited from a sample of adults aged 18 to 75 who participated in the Hong Kong Mental Morbidity Survey (HKMMS), a territory-wide study of mental illness prevalence among ethnic Chinese in Hong Kong (Lam et al., 2014). The HKMMS selected 5,700 participants based on a stratified random sampling scheme according to region and living circumstances and administered a battery of assessment instruments, including Chinese versions of the Clinical Interview Schedule–Revised (CIS-R; Lam et al., 2014; Singleton, Bumpstead, O’Brien, Lee, & Meltzer, 2001) and the Psychosis Screening Questionnaire (PSQ; Bebbington & Nayani, 1995). Based on these screening measures, 4,902 participants were identified as being free from CIS-R-defined neurotic disorders and PSQ-defined psychotic disorders.

For this study, 80 participants were randomly selected from the larger HKMMS pool of 4,902 participants. This sample size of *N* = 80 was selected to be manageable in terms of resources for this preliminary study, while sufficiently powered at 80% to detect a difference in IFES total score between HR and LR groups at an alpha level of .05. Specifically, sample size calculations based on Deeprose et al. (2011) indicated that a minimum of *n* = 18 participants in each group should be sufficient to detect a group difference on IFES total score. Given the rates of soft bipolar symptoms of 20%–25% in the general community (Lee, Ng, & Tsang, 2009; Merikangas et al., 2007), a total sample size of 80 was selected to yield sufficient numbers in each group.

These 80 participants attended in person to complete a battery of self-administered questionnaires assessing mental imagery and bipolar risk, as well as other questionnaires unrelated to this study. Participants were invited to complete the same procedure 7 weeks later. None of the target participants declined to participate at baseline but only 57 participants were traced for follow-up, representing a dropout rate of around 30%.

Participants provided written informed consent. The study was approved by the Kowloon Central and Kowloon East Cluster Research Ethics Committee of Hospital Authority of Hong Kong and was carried out in accordance with the provisions of the World Medical Association Declaration of Helsinki.

### Data collection

Except for demographic characteristics, all measures were administered twice, once at study entry (baseline) and then at the 7-week follow-up.

### Baseline demographic characteristics

A self-report demographic questionnaire yielded information on gender, age, years of education, marital status, presence of past psychiatric illness and use of psychiatric medications. The latter two were verified by checking electronic health records using the Clinical Information System of the Hospital Authority of Hong Kong.

### Clinical measures

#### Mood Disorder Questionnaire (MDQ)

The 15-item MDQ (Chung, Tso, & Chung, 2009; Hirschfeld et al., 2000; Hirschfeld et al., 2003) was used to classify participants into HR versus LR groups (Calabrese et al., 2006; Deeprose et al., 2011). A validated Chinese version was used (Chung et al., 2009). Scores of 7 or above indicated HR bipolar risk, whereas 6 and below indicated low risk. This cut-off has a sensitivity of .57 and a specificity of .82 in identifying people in the general population with bipolar spectrum disorders (Calabrese et al., 2006). Here, MDQ showed good internal consistency (Cronbach’s alpha = .83).

#### Life Events Checklist (LEC)

The Life Events Checklist (LEC; Gray, Litz, Hsu, & Lombardo, 2004; Liu, Oda, Peng, & Asai, 2007) was administered to measure experiences of potentially traumatic events (PTEs). The 17-item LEC consists of 16 items asking about multiple types of exposure to PTEs. A previously validated Chinese version of the LEC was used (Liu et al., 2007). In this study, internal consistency of the LEC was found to be good (Cronbach’s alpha = .89).

#### Clinical Interview Schedule–Revised (CIS-R)

The CIS-R (Lewis et al., 1992) is a structured psychiatric interview for assessing the presence and severity of common psychological symptoms in the past month (Singleton et al., 2001). Here, a Chinese version of the CIS-R was administered that has previously been found to be valid and reliable in diagnosing depressive and anxiety disorders (Lam et al., 2014).

#### Psychosis Screening Questionnaire (PSQ)

The Chinese version of the PSQ (Bebbington and Nayani, 1995) was found to be a valid and reliable questionnaire (Lam et al., 2014). In this study, no participants were excluded based on the PSQ.

#### Hypomanic Checklist (HCL-32)

The HCL-32 (Angst et al., 2005; Poon et al., 2012) is a 32-item self-administered questionnaire originally developed for identifying bipolarity in patients with major depression (Angst et al., 2005). In this study, the HCL-32 was included to exclude clinically hypomanic participants and to validate HR versus LR grouping of participants based on the MDQ. There is evidence that the HCL-32 has two factors: ‘active-elated hypomania’ (13 items) and ‘risk-taking/irritable hypomania’ (six items) (Angst et al., 2005). Previously, the HCL-32 has been used to identify soft bipolar symptoms among a non-clinical population, with a total score of 18 and above plus a score of 2 and above on the ‘risk-taking/irritable’ hypomania scale considered indicative of clinical hypomania (Meyer et al., 2007). In this study, a previously validated Chinese version of the HCL-32 with good sensitivity and specificity was used (Poon et al., 2012). The HCL-32 showed good internal consistency (Cronbach’s alpha = .83). In this study, no participants were excluded based on the HCL-32.

### Imagery measures

#### Impact of Future Events Scale (IFES)

The IFES (Deeprose & Holmes, 2010) is a self-report 24-item scale assessing the emotional impact of prospective imagery. Participants are asked to identify future events that they have been thinking about by imagining them over the past 7 days (i.e. prospective images) and to indicate whether each event was positive or negative in valence. As a refinement to the original IFES, and in order to increase variability on this measure, participants in this study were asked to identify an unconstrained number of future events. Subsequently, participants rated intrusive pre-experiencing, avoidance and hyper-arousal in response to their prospective images using 24 items. Examples include ‘pictures about the future popped into my mind’ and ‘I had strong feelings about the future’. Each item was rated on a scale from 0 to 4, with 0 = *not at all*, 1 = *a little bit*, 2 = *moderately*, 3 = *quite a bit* and 4 = *extremely*. An ‘IFES total’ score, which reflects the impact of prospective imagery, is calculated by summing across all 24 items, giving a total score with possible range from 0 to 96. Additionally, we created an ‘IFES total events’ score (number of events) and an ‘IFES positive events’ score (proportion of events reported as positive in valence) to test exploratory hypotheses relating to the number and valence of prospective images. The original IFES has been shown to have acceptable test–retest reliability (Pearson’s correlation = .73) and good internal consistency (Cronbach’s alpha = .87) (Deeprose & Holmes, 2010).

A Chinese version of the IFES was prepared by the first author and back-translated into English by a group of mental health professionals (two psychiatrists and two mental health nurses) blind to the purpose of the study. Any discrepancy between the original and translated versions was resolved between the first author and the translation panel. The translated IFES was then given to a group of health care workers (*N *= 20) twice over a period of 4 weeks. This showed good internal consistency (Cronbach’s alpha = .86) and good test–retest reliability (intra-class correlation = .84). In this study, internal consistency of the Chinese IFES was good (Cronbach’s alpha = .83).

#### Spontaneous Use of Imagery Scale (SUIS)

The SUIS (Reisberg et al., 2003) is a 12-item questionnaire measuring general tendency to use imagery in everyday life. Items describe use of imagery in day-to-day situations, for example, ‘when I think about visiting a relative, I almost always have a clear picture of him or her’. Participants rate each item according to the degree to which it was appropriate for them, from 1 to 5, with 1 = *never appropriate*, 3 = *appropriate half of the time* and 5 = *always completely appropriate*. Scores across items are summed to yield SUIS total score, ranging from 12 to 60, with higher total score signifying higher general tendency to use imagery in daily life. The SUIS has been used in previous studies of mental imagery in bipolar risk and bipolar disorder (Deeprose et al., 2011; Hales et al., 2011; Holmes et al., 2011).

The SUIS version was translated into Chinese and validated by the same translation panel and sample of health care workers as for the IFES (see previous section). The translated SUIS had good internal consistency (Cronbach’s alpha = .83) and test–retest reliability (intra-class correlation = .88). In this study, the SUIS showed good internal consistency (Cronbach’s alpha = .83).

### Statistical analysis

STATA-12 software was used for statistical analyses (StataCorp, 2011). Skewed continuous data were normalised using square root transformation prior to parametric analyses. Participants were split into HR and LR groups based on a predefined cut-off score of 7 at baseline on the MDQ. Categorical control variables were compared across HR and LR groups at baseline and at the 7-week follow-up using Fisher’s exact tests or Chi-squared tests. Analysis of variance (ANOVA) was used to compare continuous control variables such as age, years of education and mean LEC score across bipolar risk groups and time-points. Control variables which differed significantly between groups at either time-point were entered as covariates in subsequent analyses across groups and time-points. Tests of significance were two-tailed. Due to multiple comparisons, observed differences were considered statistically significant at a Bonferroni-corrected threshold of *p *⩽ .006 (i.e. .05/8).

To evaluate primary hypotheses regarding mental imagery susceptibility in participants at HR versus LR of developing bipolar disorder, analyses of co-variance (ANCOVA) were conducted comparing IFES total score, SUIS total score, IFES total events and IFES positive events across bipolar risk groups and time-points. Covariates were age, years of education and LEC total events, as these are theoretically important confounder variables of the relationship between mental imagery susceptibility and bipolarity (Angst et al., 2005; Holmes et al., 2008).

## Results

### Demographic and clinical variables

The whole baseline sample (*N* = 80) comprised 58 females and 22 males with a mean (*SD*) age of 45.6 (15.35) years. Seven participants at baseline reported a history of psychiatric treatment (three suffered from depression, one from mixed depressive and anxiety disorder, one from anxiety disorder, one from neurasthenia and one from insomnia) but none reported current psychiatric treatment, and based on the CIS-R and the PSQ, none was identified as suffering from current anxiety, depression or psychosis. None scored above 18 on HCL-32 total score or above 2 on the ‘risk-taking/irritable’ hypomania sub-scale, suggesting an absence of clinical hypomania (Meyer et al., 2007).

The sample was divided into HR and LR groups according to the MDQ. At baseline, the HR group consisted of *n* = 18 participants (22.5% of the total sample) and the LR group consisted of *n* = 62 participants (Table 1). At the 7-week follow-up, the HR group consisted of *n* = 13 participants (22.8% of total sample) and the LR group consisted of *n* = 44 participants. There were no significant differences between HR and LR groups at baseline or at the 7-week follow-up in gender, age, years of education, marital status, number of lifetime psychiatric illnesses or LEC traumatic events (see Table 1). Proportion of male gender, marital status and having a past history of psychiatric illness; age, years of education, baseline HCL-32 total scores and LEC total scores were similar across patients who were successfully traced for follow-up and those who could not be traced for follow-up (all *p* > .10). Missing data can thus be considered as missing at random.

**Table 1.** Demographic and baseline clinical measures in high and low bipolar risk groups (as defined by Mood Disorder Questionnaire; MDQ) at baseline (*N *= 80) and at the 7-week follow-up (*n* = 57).

Variables of interest	High bipolar risk cases		Low bipolar risk group		Statistic
	Baseline (*n* = 18)	Follow- up (*n* = 13)	Baseline (*n* = 62)	Follow-up (*n* = 44)	High vs low bipolar risk groups at baseline and at the 7-week follow-up
Male gender (%)	3 (16.7)	2 (15.4)	19 (30.6)	15 (34.1)	Baseline: Fisher’s exact test: *p* = .37; Follow-up: Fisher’s exact test, *p* = .30
Mean age (*SD*)	41.6 (13.61)	46.0 (15.46)	46.8 (15.73)	45.5 (16.07)	ANOVA Group × time; *F*(156) = .59, *p* = .59
Years of education (*SD*)	14.3 (5.27)	15.6 (4.49)	13.3 (4.81)	13.3 (5.29)	ANOVA Group × time; *F*(156) = .02, *p* = .96
Marital status					
Single or divorced (%)	8 (44.4)	7 (53.8)	22 (35.5)	15(34.1)	Baseline: *x*^2^ = 1.24, *p* = .27;
Married/cohabiting (%)	10 (55.6)	6 (46.2)	40 (64.5)	29 (65.9)	Follow-up: *x*^2^* *= 3.14, *p *= .08
Presence of past psychiatric illness (%)	2 (11.1)	2(15.4)	5 (8.1)	4 (9.1)	Baseline: Fisher’s exact test, *p *= .65; Follow-up: Fisher’s exact test, *p *= .61
Mean LEC-total score (*SD*)	.9 (1.28)	1.0 (1.41)	.6 (.91)	.6 (.87)	Group × time *F*(156)* *= 1.04, *p *= .30
Mean HCL-32 total score (*SD*)	18.3 (3.44)	17.4 (3.63)	12.9 (4.53)	12.3 (4.21)	Group × time: *F*(156)* *= .12, *p *= .73; Risk group: *F*(156)* *= 23.5, *p* < .001 Time: *F*(156)* *= .39, *p *= .54

### Group differences in HCL-32 score

Providing convergent validity to the grouping of participants into HR and LR based on the MDQ, ANOVA comparing mean HCL-32 total scores across risk group and time showed a main effect of Group and no interaction with Time (Group: *F*(152)* *= 23.5, *p* < .001; Group × Time; *F*(152)* *= .12, *p *= .13), with higher HCL-32 total scores in HR than LR at baseline and at the 7-week follow-up (Table 1).

### Mental imagery susceptibility across bipolar risk groups and time

Repeated measures ANCOVA comparing IFES total score across HR versus LR groups at baseline and follow-up showed no significant Group × Time interaction (Table 2). Consistent with the first hypothesis, there was a main effect of risk group, with higher IFES total scores in HR than LR at baseline and at follow-up. Consistent with the second hypothesis, there was no main effect of time. In terms of the covariates, years of education had no effect on IFES total score (*F* [152]* *= .11, *p *= .74), although there was a marginal effect of age (*F* [152]* *= 10.8, *p *= .002) and number of traumatic events (*F* (152)* *= 6.70, *p *= .01).

**Table 2.** Mental imagery characteristics between high bipolar and low bipolar risk groups (as defined by Mood Disorder Questionnaire; MDQ) at baseline (*N *= 80) and at the 7-week follow-up (*n *= 57).

Variables of interest	High bipolar risk		Low bipolar risk		Statistics: repeated measures ANCOVA^a^
	Baseline (*n *= 18)	Follow-up (*n *= 13)	Baseline (*n *= 62)	Follow-up (*n *= 44)	
Mean (*SD*) IFES total	32.8 (12.50)	36.4 (14.60)	23.3 (10.69)	22.6 (9.21)	Group × time interaction: *F*(152)* *= 1.76, *p *= .19; Main effect (group): *F*(152)* *= 13.08, *p *= .001; Main effect (time): *F*(152)* *= .06, *p *= .81)
Mean (*SD*) SUIS* total	40.4 (6.90)	44.1 (9.34)	32.2 (9.15)	35.6 (9.07)	Group × time interaction: *F* (152)* *= .02, *p *= .90; Main effect (group): *F*(152)* *= 12.90, *p *= .001; Main effect (time): *F* (152)* *= 1.81, *p *= .19)
Mean (*SD*) IFES number of events	2.5 (.78)	2.5 (.78)	2.7 (.67)	2.4 (.87)	Group × Time interaction: *F*(152)* *= 3.44, *p *= .07; Main effect (group): *F*(152)* *= .04, *p *= .84; Main effect (time): *F* (152)* *= 2.86, *p *= .10)
Mean (*SD*) IFES proportion of positive events	.7 (.32)	.7 (.35)	.8 (.25)	.8 (.31)	Group × time interaction: *F* (152)* *= .17, *p *= .68; Main effect (group): *F* (152)* *= 2.39, *p *= .13; Main effect (time): *F* (152)* *= .22, *p *= .64)

IFES = impact of future events scale; SUIS = spontaneous use of imagery scale.

a ANCOVA: repeated measures analysis of co-variance was conducted using the following variables as covariates – age, years of education and square root transformed values of LEC total scores.

Repeated measures ANCOVA comparing SUIS scores across HR versus LR at baseline and follow-up showed no Group × Time interaction (Table 2). Consistent with the first hypothesis, there was a main effect of Group, with higher SUIS scores in HR than LR at baseline and at follow-up. There was no main effect of time. None of the covariates was individually significant.

Repeated measures ANCOVA comparing IFES number of events across HR versus LR at baseline and follow-up showed no significant Group × Time interaction, no main effect of group and no effects of the covariates (see Table 2).

Repeated measures ANCOVA comparing IFES proportion of positive events across HR versus LR at baseline and follow-up showed no significant Group × Time interaction and no effect of time and no effects of the covariates (Table 2).

### Post hoc regression analysis

To understand the presence of any possible dose–response relationship between mental imagery susceptibility and bipolarity, a post hoc hierarchical linear regression analysis was performed predicting MDQ total score based on mental imagery and other variables. Gender, age, years of education and the number of lifetime traumatic events were entered in Block 1 as they were considered as theoretical confounders of mental imagery susceptibility and bipolarity. SUIS total scores, IFES total scores and IFES total event scores were entered in Block 2. The overall model was significant (*R*^2^ = .27, *F*(870) = 3.18, *p* = .004), with IFES total score the only significant predictor of MDQ total score (*B* = .08, *SE* = .03, Beta = .31, *t* = 2.63, *p* = .01).

## Discussion

This study is the first to examine the relationship between mental imagery susceptibility and bipolar risk in a general community sample of Chinese adults screened to be free from major psychiatric disorders, that is, a random sample of participants recruited from the general population. This is also the first study to examine stability in mental imagery characteristics longitudinally over a period of 7 weeks in participants identified as being at high risk of developing bipolar disorders. Findings may have relevance for understanding the possible role of mental imagery susceptibility in emotional instability and in bipolar disorders.

### General use of imagery in daily life, emotional impact of prospective imagery and bipolar risk

Participants identified as being at high risk of developing bipolar disorders reported higher levels of general use of imagery in daily life (SUIS) and experienced greater emotional impact of prospective imagery (IFES) than did those identified as being at LR. This is the first study that has confirmed that such differences in imagery characteristics between HR and LR groups are present in a general community sample, and also in people from a non-Western population. Such findings suggest that enhanced mental imagery susceptibility among people with HR generalises cross-culturally.

Combined with some recent evidence that mental imagery susceptibility may be elevated in patients with bipolar disorder compared to patients with unipolar depression or healthy controls (Hales et al., 2011; Holmes et al., 2011), these results suggest that mental imagery susceptibility (in terms of general use of imagery and emotional impact of prospective imagery) might be a possible vulnerability factor associated with emotional instability in bipolar disorders.

It is interesting that when bipolarity was considered as a continuous variable (in a post hoc analysis recommended by reviewers), our regression modelling indicated that the emotional impact of prospective imagery (IFES total) was the strongest predictor of MDQ total score. Prospective mental imagery may be a particularly good target for future research.

### Stability over time

This study examined whether mental imagery characteristics and their relationship to bipolar risk status fluctuate spontaneously over time by testing participants twice, 7 weeks apart. Results indicate stability over time in mental imagery susceptibility. This finding is consistent with the hypothesis that mental imagery susceptibility may be a stable characteristic of individuals scoring highly on measures of hypomania such as the MDQ. In future research, prospective studies are needed to investigate, over a longer period of time, any causal relationship between mental imagery susceptibility and risk for developing bipolar disorders, for example, in the event of a life stressor (cf. Malik et al., 2014).

### Strengths and limitations

This study has several strengths. First, it included Chinese adults selected at random from a larger representative community sample (*N* = 5,700) from a population-wide mental health survey. There is evidence for more stigma and negative attitudes towards mental health problems in Chinese than Western populations, resulting in possible under-reporting of hypomanic symptoms (Poon et al., 2012) and a lower prevalence of bipolar disorders among the Chinese population (Phillips et al., 2009). However, to date, studies of mental imagery have been conducted in predominantly Western student samples.

Second, the sample was screened to be free from important potential clinical confounding variables, such as depressive or anxiety disorders and psychotic disorders (CIS-R, Lewis et al., 2002; PSQ; Bebbington & Nayani, 1995) and presence of traumatic life events (LEC; Gray et al., 2004), thus controlling for potential confounders of the putative relationship between imagery and bipolar risks.

Third, the prospective design showing persistently elevated mental imagery among HR versus LR groups provides new evidence in support of the notion that mental imagery susceptibility may be a vulnerability marker associated with bipolarity or emotional instability (Holmes et al., 2008).

Fourth, higher total scores on the HCL-32 (severity of past hypomanic symptoms) in the HR versus LR groups strengthens the validity of the MDQ criterion cut-off score used for defining these groups. At the same time, the absence of clinical hypomania in the HR group may indicate heightened imagery susceptibility that contributes to emotional instability rather than being a consequence of clinical hypomania.

Finally, Chinese versions of established instruments used for measuring mental imagery susceptibility, mood symptoms and bipolar risk status were developed and validated locally (Chung et al., 2009; Poon et al., 2012), bolstering validity of the findings.

This study suffers from a number of limitations. Although the choice of sample size was based on a priori sample size calculations, it is possible that type II errors occurred, missing clinically significant differences between the two bipolar risk groups on the total number of general and positive prospective images. Second, there was a dropout rate of 30% at the 7-week follow-up, which might lead to potential attrition bias. However, those who were successfully traced for follow-up and those who could not be traced were not significantly different in major demographic and clinical variables, suggesting that this limitation does not apply to this study. Third, the major outcome variable (bipolar risk) and predictor variables (mental imagery measures) were based on self-administered questionnaires, as is common in epidemiological studies, rather than structured clinical interviews or laboratory measures. Finally, the random adult age sample might have led to selection bias for people with risk of conversion to late-onset bipolar disorder, which may be clinically and genetically different from those with early-onset bipolar disorder (Leboyer, Henry, Paillere-Martinot, & Bellivier, 2005).

## Clinical implications and conclusion

This study shows preliminary evidence for heightened mental imagery susceptibility across a period of 7 weeks in individuals identified as being at HR versus LR of developing bipolar disorders, drawn from a representative community sample of Chinese adults. The above findings thus provide further support for the notion that mental imagery characteristics (general use of imagery of daily life; emotional impact of prospective imagery) might be cognitive risk factors associated with bipolarity. It is possible that heightened imagery susceptibility contributes to emotional instability rather than being a consequence of clinical onset of bipolar disorders or bipolarity. We suggest that the occurrence of prospective imagery may provide fuel to trigger emotional instability in those at HR (Holmes et al., 2008; Ivins et al., 2014). The study provides preliminary evidence that mental imagery susceptibility could be a cognitive marker worth exploring in future studies with larger sample sizes and high familial risks for bipolar disorders and with longer follow-up periods. Investigating mental imagery (in addition to verbal thoughts) may be useful in developing psychological treatments for bipolar disorder, for example, in the area of Cognitive Behavioural Therapy (Ng et al., 2013; Stratford, Cooper, Di Simplicio, Blackwell, & Holmes, 2015), and in psychiatry more generally (Di Simplicio, McInerney, Goodwin, Attenburrow, & Holmes, 2012). This study provides just one step in that direction.
